# A benchmark dataset and case study for Chinese medical question intent classification

**DOI:** 10.1186/s12911-020-1122-3

**Published:** 2020-07-09

**Authors:** Nan Chen, Xiangdong Su, Tongyang Liu, Qizhi Hao, Ming Wei

**Affiliations:** Inner Mongolia Key Laboratory of Mongolian Information Processing Technology, College of Computer Science, Inner Mongolia Univeristy, University West Road, Hohhot, China

**Keywords:** Intent classification, Dataset, Word segmentation, Name entity recognition

## Abstract

**Background:**

To provide satisfying answers, medical QA system has to understand the intentions of the users’ questions precisely. For medical intent classification, it requires high-quality datasets to train a deep-learning approach in a supervised way. Currently, there is no public dataset for Chinese medical intent classification, and the datasets of other fields are not applicable to the medical QA system. To solve this problem, we construct a Chinese medical intent dataset (CMID) using the questions from medical QA websites. On this basis, we compare four intent classification models on CMID using a case study.

**Methods:**

The questions in CMID are obtained from several medical QA websites. The intent annotation standard is developed by the medical experts, which includes four types and 36 subtypes of users’ intents. Besides the intent label, CMID also provides two types of additional information, including word segmentation and named entity. We use the crowdsourcing way to annotate the intent information for each Chinese medical question. Word segmentation and named entities are obtained using the Jieba and a well-trained Lattice-LSTM model. We loaded a Chinese medical dictionary consisting of 530,000 for word segmentation to obtain a more accurate result. We also select four popular deep learning-based models and compare their performances of intent classification on CMID.

**Results:**

The final CMID contains 12,000 Chinese medical questions and is organized in JSON format. Each question is labeled the intention, word segmentation, and named entity information. The information about question length, number of entities, and are also detailed analyzed. Among Fast Text, TextCNN, TextRNN, and TextGCN, Fast Text and TextCNN models have achieved the best results in four types and 36 subtypes intent classification, respectively.

**Conclusions:**

In this work, we provide a dataset for Chinese medical intent classification, which can be used in medical QA and related fields. We performed an intent classification task on the CMID. In addition, we also did some analysis on the content of the dataset.

## Background

In the early years, users mainly employ search engines to obtain answers to medical questions [1]. They enter keywords in the search box and get many pages involving the keywords. However, it is difficult for users to filter out irrelevant information and judge the correctness when a considerable amount of information is available. Although more and more sophisticate retrieval and sorting methods were proposed, it is still an overload for users to extract the expected answers from massive web pages.

With the development of knowledge representation, knowledge graph-based medical QA systems gradually attract more and more attention [2, 3]. They not only allow users to ask medical questions in natural language but also returns accurate answers to users without answer selection anymore. This offers great convenience for people’s access to health knowledge.

The process of knowledge graph-based medical QA [4] systems can be divided into three steps when using a knowledge graph based medical QA systems, including intent understanding [5], answer retrieval [6] and answer generation [7, 8]. Intent understanding is to analyze what the users want and plays a core role in the whole answer process. It can be treated as a classification problem if we restrict the intended scope in advance. Traditional methods of intent classification include keyword matching [9], template matching [10], etc. The disadvantage of these methods is their poor generalization ability. Recently, more deep-learning-based approaches gradually attract more and more attention and have achieved excellent performance in intention classification.

In the field of medical QA, there is a demand to understand the user’s question accurately before answer generation. Besides, compared with English, Chinese has more diverse expressions. Therefore, we should introduce intent comprehension to refine the areas of Chinese medical questions further to provide better answers. For Chinese medical intent understanding, it requires a high-quality dataset to train a deep-learning-based approach in a supervised fashion. However, such a dataset is unavailable for Chinese medical intent understanding, and other areas datasets for intent understanding are not suitable for the medical QA system.

To solve the above problem, we constructed a Chinese medical intent dataset (CMID) using the questions from medical QA websites [11]. The questions in CMID data are obtained from several medical QA websites. The intent annotation standard is developed by the medical experts, which includes four types and 36 subtypes of users’ intents. Besides the intent label, CMID also provides two types of additional information, including word segmentation and named entity. We use the crowdsourcing way to annotate the intent information for each Chinese medial question. Word segmentation and named entities are obtained using the Jieba [12] and a well-trained Lattice-LSTM model [13]. To obtain more accurate results, we loaded a Chinese medical dictionary consisting of 530,000 medical terms for word segmentation. Finally, we select four popular deep-learning-based models and compare their performances of intent classification on CMID.

The contributions are mainly as follows:

- We provide a large-scale, high-quality Chinese medical question dataset which contains the intent, word segmentation, and name entities labels. It can be used in the intent classification of the Chinese medical QA system. We will publish the dataset CMID at http://www.github.com/liutongyang/CMID.
- We compared four deep learning based models for intent classification on CMID and found the best model for Chinese medical intent classification.

## Methods

### Constructing the dataset CMID

As mentioned above, the questions in CMID have labeled the intention, word segmentation, and named entity information. All questions were collected from 20 online professional medical QA websites. On these websites, users can ask such a variety of questions about medical matters in Chinese, and doctors provide professional diagnosis and advice under the questions. We extract the questions from the web pages using regular expression and manually annotate the intent types through crowdsourcing. Automatic tools fulfill word segmentation and named entities of the questions. The processing workflow is as follows:

- **Creating the standard:** Before getting into the details of how to annotate the questions, we need to create a standard of medical intent. In the project of CMID, the intent annotation standard is developed by the medical experts, which includes four types and 36 subtypes of users’ intents. It details the definition of each intent type and the guidelines of how to deal with the annotation inconsistency. Besides the intent label, CMID also provides two types of additional information, including word segmentation and named entity. The entity tags fellow the classification standard of the ccks2019 evaluation task1.
- **Data preprocessing:** We first crawl the web pages from several Chinese medical QA websites. Then, we use regular expressions to eliminate all emoticons, garbled, HTML code, and hyperlinks, leaving only numbers, punctuation, Chinese, and English characters. To avoid data bias, we removed the questions whose lengths are less than four words and more than 255 words at last. In order to ensure the balance of the data, our original data is equally selected according to departments, and repeated questions are removed.
- **Intent annotation:** We adopt crowdsourcing to annotate the intent type for the questions in CMID manually. The advantage of crowdsourcing involves taking a tremendous job and breaking it into many smaller jobs that a crowd of people can work on separately. According to the annotation standard, the user’s intent is divided into four types, including “disease,” “medicine,” “therapeutic schedule,” and “other.” Each type is further divided into several subtypes. According to the experience of experts, more additional information can help understand the intent of the question. We also provide the annotator with department information and doctor’s answers to each question to facilitate intentional discrimination. Each question is annotated by two people simultaneously. If there is a conflict, we resort to the medical experts. Due to the particularity of the medical field, labelers should also have medical expertise. This limits the number of labelers and annotation progress. The resulting CMID contains 12000 questions in total.
- **Word segmentation:** To provide additional useful information, we use Jieba (https://github.com/fxsjy/jieba) to segment the sentences in precise mode, which is one of the three modes of Jieba. It can produce a more accurate result at the cost of more running time. In word segmentation, it is difficult to distinguish the word boundaries of Chinese medical terminology. To ensure that terminology can be accurately segmented, we loaded a medical dictionary containing 534,983 medical terms for the Jieba.
- **Named entity recognition:** For the QA task, the named entities in questions can provide vital clues for answer generation. Therefore, we label the named entities for the questions in CMID. In this project, we use Lattice-LSTM for entity recognition, which is the best performing model on the Chinese named entity recognition task. It can take full advantage of input character and word order information without word segmentation errors. The inputs are single Chinese character embeddings and word embeddings, and the outputs are the entity labels.

### Text classification model

In this paper, we offer a case study of intent classification on CMID. Four deep-learning-based models were compared, including TextCNN [14], TextRNN [15], Fast Text [16], and TextGCN [17]. We used default parameter settings as their papers or open-source implementations. For TextCNN and TextRNN, we used pre-trained word embeddings provided by Tencent-AILab [18].

- **TextCNN:** The feature of CNN is the shared convolution kernel, which can be paralleled in the calculation, thus significantly reducing the time for model training. We use the traditional CNN structure to extract text information, which consists of an input layer, convolution layer, pooling layer, and fully connected layer. Firstly, the sentence enters the input layer through the word segmentation, and then the input layer embeds the word into the word vector. In the convolutional layer, we use four different convolution kernel sizes to extract information. The width of the convolution kernel is the length of the word vector, 200 dimensions. The length of the convolution kernel is set to 1, 2, 3, 4, respectively, corresponding to the single word feature, the 2-gram feature, the 3-gram feature, and the 4-gram feature in the sentence. In the pooling layer, we use max-pooling [19] to process the data and concatenate the four features. Finally, we output the most probable category as the final prediction.
- **TextRNN:** The advantage of RNN is capturing longer distance dependencies in the sequence. It is unnecessary to adjust the cumbersome hyperparameters like CNN. We use bidirectional LSTM [20] to capture the context information of the sentence entirely. Firstly, the sentences are mapped into the word vector after being segmented. The input layer then enters the words into the model in chronological order. In order to improve the robustness of the model, we add the dropout operation in each layer of LSTM. Finally, the model takes the hidden state of the last layer of LSTM as the input to the fully connected softmax layer and obtains the probability value for each category. We output the category with the highest probability value as the result of the final prediction.
- **Fast Text:** Fast Text was Facebook’s open-source text classification model in 2016. Its model structure is very similar to the CBOW model structure in word2vec [21, 22]. There are three layers in the Fast Text model, the input layer, the hidden layer, and the output layer. First, each question is segmented, and then its N-gram feature is used as the input layer, embedding in the hidden layer and averaged to get the hidden layer output. Finally, the maximum probability label is calculated by the hierarchical softmax classifier. Due to its simple model structure, Fast Text is very fast in training compared to other neural network models. At the same time, it has achieved high classification accuracy and is widely used in the industry. Therefore, we use Fast Text as one of the intent classification models on CMID.
- **TextGCN:** TextGCN builds a large and heterogeneous text graph on the entire corpus that contains word nodes and document nodes. It can explicitly model the global word co-occurrence. When the model is initialized, the feature matrix only needs to be set to an identity matrix. In other words, each word or document is input to the TextGCN as a one-hot vector. There are two types of edges in the graph, document-word is constructed with TF-IDF information, and word-word is constructed with point-wise mutual information (PMI) [23]. When the text graph is built, we feed the graph into a simple two-layer GCN, such as Kipf’s work [24]. In the hidden layer, the node and the neighbor node represent each other to complete the information transfer, and finally, send it into a softmax classifier to calculate the maximum probability label.

## Results

### The dataset CMID

In CMID, there are four types and 36 subtypes of intent labels, as listed in Table 1. The descriptions of the subtypes are also provided.

**Table 1:** Four types and 36 subtypes classification of CMID

Type	Subtype	Description
Disease	Definition	Definition of disease
Disease	Pathogeny	Causes of disease
Disease	Clinical manifestation	Manifestations of the disease
Disease	Related diseases	Sequelae, complications, etc
Disease	Treatment method	Treatment of disease
Disease	Recommended hospital	Good at treating the disease
Disease	Prevention	How to prevent disease
Disease	Subordinate departments	Belongs to which departments
Disease	Disease taboo	What should not be done
Disease	Infectivity	Whether the disease is contagious
Disease	Cure rate	Whether it can be cured
Disease	Severity	Severity of disease
Medicine	Effect	Role of medicine
Medicine	Applicable disease	Which diseases are suitable for
Medicine	Price	Medicine price
Medicine	Drug contraindication	Conflict with drug
Medicine	Usage	Drug usage
Medicine	Side effect	Side effects of drugs
Medicine	Ingredient	Pharmaceutical ingredients
Treatment programs	Method	Method of the operation
Treatment programs	Cost	Cost of the operation
Treatment programs	Effective time	Effective time after the operation
Treatment programs	Inspection purpose	X-ray, CT, etc
Treatment programs	Treatment time	Time of the operation
Treatment programs	Curative effect	Operation effect
Treatment programs	Recovery Time	Recovery time after the operation
Treatment programs	Normal indicator	Normal indicators of the operation
Treatment programs	Physical examination program	Including various checks
Treatment programs	Recovery	recover health after the operation
Other	Device usage	Use of various instruments
Other	Multi-questions	More than one question

**Table 1:** Four types and 36 subtypes classification of CMID (*Continued*)

Type	Subtype	Description
Other	Health preservation	All about health preservation
Other	Cosmetic surgery	All about Cosmetic surgery
Other	Gender issues	All about Gender issues
Other	Contrast	Medicine comparisons, hospital,etc
Other	Uncertainty	Custom intentions

To facilitate user’s access and modification, the dataset CMID is stored in JSON format. The field "questions" stores the user’s question. The field "entities" stores all the entities in the sentence together with their types (“label-type”), the starting position (“start-pos”), and the ending positions (“end-pos”) of the entity in the sentence. The field “seg-result” represents the result of the word segmentation. The field “intent” is the manually annotated intent label. Table 2 presents an example in CMID.

**Table 2:** An example in CMID

Key	Value	Example
question	user question	
entities	entity extraction results	[“label-type”: “Desease”, “start”: 0, "end": 5]
seg-result	word segmentation results	["“, “”, ““, “, ?”]
intent	intent label of the question	Definition

According to statistical analyses, we list intent type and subtype information of CMID in Tables 3 and 4, respectively. Table 3 reports the number of each intent type, the maximum, the minimum, and the average length of the questions in each type. Table 4 is similar to Table 3.

**Table 3:** Details of four types of intent classification

Type name	Number	Max length	Min length	Average length
Disease	6264	217	5	30
Medicine	2755	205	4	27
Treatment programs	904	213	5	32
Other	2332	221	5	37

**Table 4:** Details of 36 subtypes of intent classification

Subtype name	Number	Max length	Min length	Average length
Definition	440	131	5	24
Pathogeny	970	157	5	26
Clinical manifestation	1313	217	5	32
Related diseases	242	139	8	33
Treatment method	2101	185	5	30
Recommended hospital	337	146	8	43
Prevention	138	111	6	29
Subordinate departments	26	60	6	22
Disease taboo	271	122	7	26
Infectivity	43	111	5	42
Cure rate	144	135	6	36
Severity	263	107	7	40
Effect	555	205	6	27
Applicable disease	962	110	7	28
Price	75	109	9	31
Drug contraindication	235	102	7	28
Usage	420	142	6	28
Side effect	332	88	8	29
Ingredient	104	83	5	25
Method	423	213	5	33
Cost	86	102	6	42
Effective time	31	68	10	26
Inspection purpose	45	163	9	36
Treatment time	41	119	9	48
Curative effect	64	87	5	26
Recovery Time	30	89	9	29
Normal indicator	24	72	10	26
Physical examination program	80	107	7	29
Recovery	80	117	9	30
Device usage	20	121	10	38
Multi-questions	1132	221	6	45
Health preservation	139	87	5	26
Cosmetic surgery	8	36	6	17
Gender issues	84	127	5	31
Contrast	114	100	9	26
Uncertainty	835	196	5	31

### Intent classification result on CMID

In this paper, we carried out a case study on CMID to explore the performance of the deep learning-based models on Chinese medical intent classification. Two group experiments were implemented for four intent types and 36 subtypes, respectively. The training set and test set are divided by a ratio of 4:1 according to each category.

The evaluation metric is the accuracy *p*. The reason we focus on accuracy is that the accuracy of the intent classification plays a decisive factor in the subsequent steps of the medical QA task. The more accurate that the intent classification is, the better that the system performs. The accuracy *p* is defined as follows:

1 $$\begin{array}{@{}rcl@{}} P=\frac{T}{(T+F)} \end{array} $$

where *T* is the number that predicts the correct labels in the test set, *F* is the number that predicts the error labels in the test set.

In the experiment, the deep-learning models include TextCNN, TextRNN, Fast Text, and TextGCN. They have shown very competitive performance in text classification tasks on other datasets. The intent classification result of four types on CMID is shown in Table 5, and that of 36 subtypes is shown in Table 6.

**Table 5:** Four types intent classification result

Model	Accuracy
TextCNN	0.859
TextRNN	0.814
Fast Text	0.860
TextGCN	0.683

**Table 6:** 36 subtypes intent classification result

Model	Accuracy
TextCNN	0.473
TextRNN	0.424
Fast Text	0.462
TextGCN	0.357

From Tables 5 and 6, we can clearly see that Fast Text performs the best among the four models on Chinese medical intent classification. The performance of TextGCN is worse than any other model.

## Discussion

### The discussion of the intent classification result

From the intent classification results, we can see that the results of the four types intent classification are significantly better than the 36 subtypes intent classification, and the accuracy of the four types intent classification is more than 80%, but the results of the 36 subtypes intent classification are relatively poor. There are two reasons for this result. First, the four types of intent classification models are easier to train than the 36 subtypes’ intent classification model. Second, because the number of each category in the four types is large, the data of each category can be fully trained. The number of each category in the 36 subtypes is relatively small, resulting in a reduction in accuracy. Therefore, augmenting the data set is very important for intent recognition.

For the intent classification model, TextGCN performs worst of all models. Fast Text and TextCNN achieve the best accuracy in 4 types, and 36 subtypes classification tasks, respectively, and their results in each classification task are very close. The Text RNN has a large decrease in accuracy compared to them. This is because the average length of the questions is 30 characters, which is a short text. TextCNN and Fast Text are more powerful than TextRNN in short text feature extraction.

### The discussion of dataset content

Based on the CMID, we also did some analysis on the content of the dataset. The first noteworthy is that in CMID, 52% of the intentional label is "disease," and further, the "treatment method" accounts for 33% of all disease categories. When we delved into the question of such intentions, we found an interesting thing. 30% of users’ intentions are not clear. For example: “,,”,“, ,,,,,”. As we have seen, these two sentences have no obvious intentional keywords "why," "how to do" or "how to treat," etc. Their problem is only to describe the condition, but the patient wants to ask the doctor how to treat. Obviously, methods based on template matching and keywords are unable to obtain the semantics of such sentences. However, some patients believe that such a questioning method is effective. Therefore, we believe that in the pipeline of the intelligent medical QA system, it is necessary to train an intent recognition model.

The second thing worth discussing is that we find patients not only describe the condition when asking for treatment but at any time. In CMID, 65% of patients like to describe the condition first, and then show the intention, for example, “, ”,“, , , , , ? () , , ?”. Obviously, the intent of the previous sentence is the applicability of the drug, but the first half of the sentence is describing the illness, and the task of intent classification is invalid information. The intent of the latter sentence is the contraindication of the drug. The first half of the sentence is still describing its symptoms, which is invalid information for the intended classification task. We suggest all authors who use this dataset: it is necessary to include location information in the model when text categorization, paying particular attention to the tail of the sentence, with 65% of real user intent appearing here.

Thirdly, in all the categories, we define the “Uncertainty” category in the 36 subtypes as Out-of-scope data, and it accounts for 6% of the CMID. We think it is necessary to set Out-of-scope data to ensure the robustness of the QA system. For this phenomenon, we suggest that the QA system add multiple rounds of dialogue to determine user intent further.

Finally, there is a bit of thinking about a special intent called "multiple questions." In our intent category, there is a type of problem that we call "multiple questions," that is, a question contains two or more intents. For example: “? ? ? ? ? ?”, “, ? ? ?”. We recommend that when dealing with such sentences in a real-life scenario, supervised automated methods are used to complement the subject of the sub-question. Finally, the questions are divided into independent sub-problems and treated as complete problems.

## Conclusions

In this paper, we construct CMID, which is a large-scale, high-quality question-intentional annotation dataset for the QA system and a variety of applications in related fields. With the help of expert knowledge, we extracted four types and 36 subtypes of question intent in the real medical QA scene, and finally got 12000 questions with tag information through crowdsourcing. Besides, we also provided accurate word segmentation information and named entity recognition information. We used word-based input to evaluate the performance of four text classification baseline models. The experimental results show that the Fast Text is superior to other models. Through these experiments, we provided a strong benchmark dataset for the intent of understanding the task of medical QA. We hope that our research and dataset will promote the development of smarter, more powerful medical QA systems.

## Data Availability

The dataset is publically available via http://www.github.com/liutongyang/CMID.

## Abbreviations

TF-IDF: Term frequency-inverse document frequencies
QA: Question and answer
NER: Named entity recognition
GCN: Graph convolution neural network
CNN: Convolutional neural networks
RNN: Recurrent neural network
LSTM: Long short-term memory
